# Comorbidity of Pain and Depression in a Lumbar Disc Herniation Model: Biochemical Alterations and the Effects of Fluoxetine

**DOI:** 10.3389/fneur.2019.01022

**Published:** 2019-09-24

**Authors:** Lun Cai, Qianchao He, Yongjing Lu, Yuying Hu, Wei Chen, Liping Wei, Yueqiang Hu

**Affiliations:** ^1^Department of Neurology, The First Affiliated Hospital of Guangxi University of Chinese Medicine, Guangxi University of Chinese Medicine, Nanning, China; ^2^Department of Nuclear Medicine, Minzu Hospital of Guangxi, Nanning, China; ^3^Department of Rehabilitation Medicine, The First Affiliated Hospital of Guangxi University of Chinese Medicine, Guangxi University of Chinese Medicine, Nanning, China

**Keywords:** lumbar disc herniation, mechanical allodynia, thermal hyperalgesia, depression, serotonin, TNF-α, taste preference test, forced swim test

## Abstract

**Summary of Background Data:** Depression is one of the most common comorbidities in patients with chronic low back pain. However, the mechanisms of depression in chronic low back pain patients and the effect of antidepressants on the comorbidity of pain and depression need to be further explored. The establishment of the appropriate animal models and of more effective therapies is critical for this comorbidity. Lumbar disc herniation (LDH) is the most common disease that causes low back pain. The current study examined whether an LDH model shows behavioral and biochemical alterations that are in accordance with the characteristics of the comorbidity of pain and depression and tested the effect of fluoxetine (FLX) on these measures.

**Objective:** The current study examined whether an LDH model showed the behavioral and biochemical alterations that were in accordance with the characteristics of the comorbidity of pain and depression and tested the effect of FLX on these measures.

**Methods:** The LDH animal model was generated by the implantation of the autologous nucleus pulposus on the left L5 nerve root just proximal to the dorsal root ganglion in Wistar rats. Pain intensity was evaluated by mechanical allodynia and thermal hyperalgesia, and changes in depressive behavior were examined by the taste preference and forced swim tests. Hippocampal serotonin (5-HT) levels were measured by liquid chromatography-mass spectrometry, and tumor necrosis factor-α (TNF-α) mRNA was quantified using real-time reverse transcriptase PCR.

**Results:** LDH resulted in chronic pain, which further induced depressive behavior that persisted for 6 weeks after surgery. There were decreased 5-HT concentrations and upregulated TNF-α mRNA levels that were accompanied by behavioral changes. FLX treatment improved depressive behavior and moderately alleviated pain through increased 5-HT concentrations, and inhibited TNF-α mRNA expression.

**Conclusions:** In summary, our studies provide initial evidence that the LDH chronic pain model might serve as a model of the comorbidity of low back pain and depression. The finding that FLX improved depressive behavior and pain through normalized 5-HT concentrations and TNF-α mRNA expression establishes the initial mechanism of the comorbidity of pain and depression.

## Introduction

Depression represents one of the most common comorbidities in patients with chronic low back pain (LBP) (1). LBP contributes to low health-related quality of life (HRQoL) scores and higher pain scores, lower labor productivity and increased healthcare use (2), and the psychological symptoms, and pain-related disability may further reduce the probability of recovery from chronic LBP (3). Currently, antidepressants are suggested for symptomatic management (4). However, the mechanisms of depression in chronic LBP patients are not clear. Establishing and evaluating animal models of this comorbidity is the basis for both understanding the mechanisms and identifying appropriate treatments. However, we did not find any reports of lumbar disc herniation (LDH) animal models as a model of the comorbidity of pain and depression. The current study examined whether the LDH model was in accordance with the characteristics of the comorbidity of pain and depression and tested the effect of fluoxetine (FLX).

Comorbidity of pain and depression has long been recognized. But the underlying basis for this comorbidity is complex. Most studies have focused on hypothalamic-pituitary-adrenal (HPA) axis, neurogenesis, various neurotransmitters/neuromodulators, including serotonin (5-HT), norepinephrine (NA), dopamine (DA), glutamate (GLU), and GABA, the endocannabinoid system, brain-neurotrophic factor, opioids, neuroinflammatory factors (5, 6). Among this molecular neurobiology of the comorbidity, neuroinflammatory pathogenic mechanisms (7, 8), and 5-HT deficiency (9, 10) were widely investigated.

Central 5-HT deficiency is not only a mechanism of depression but also a mechanism of pain. 5-HT is involved in nociceptive pain signaling via ascending and descending modulatory pathways (11). The dysfunction of serotonergic transmission urges the use of particular drugs, namely, selective serotonin reuptake inhibitors (SSRIs). FLX was the first SSRI on the market; when it is administered, the inhibition of presynaptic serotonin transporters (5-HTT) causes less 5-HT to be transported from the synapse into pre-synaptic neurons, which increases the amount of 5-HT present in the synapse (12). It was used to uncover a pathophysiological link between pain and depression (13). Besides 5-HT deficiency, procytokines are an important pathogenic mechanism for depression through the activation of oxidative and nitrosative stress pathways, and the induction of indoleamine 2,3-dioxygenase (IDO) expression, which contributes to depression (14). Hippocampal procytokines are also an important pathogenic mechanism for pain through the induction of IDO and the inhibition of the melatoninergic system (15). Furthermore, the effect of FLX on the above-mentioned pathogenic mechanisms of comorbidity was tested. Previous studies concluded that FLX was effective to normalize BDNF (16, 17), enhance neurogenesis (18, 19), and inhibit GLU transmission (20, 21). Besides these, the effects of FLX on DA, NE, and 5-HT neurotransmission (22, 23) as well as neuroinflammatory factors (7, 8) were widely verified. Therefore, in this study, we also investigated the relationship between 5-HT levels, TNF-α mRNA expression, and the comorbidity of pain and depression and the effect of FLX on these measures.

## Materials and Methods

### Animals

Sixty- to sixty-five-day-old male Wistar rats (Shanghai Laboratory Animal Center, Shanghai) were housed and handled in strict accordance with the guidelines of the institutional and national Committees of Animal Use and Protection. Rats were housed in a temperature— (23 to 25°C) and humidity— (45 to 55%) controlled environment with a 12/12-h modified dark-light cycle (lights on from 7.00 a.m. to 7.00 p.m.). The protocol was approved through the Committee on the Ethics of Animal Experiments of the Guangxi University of Chinese Medicine (Permit Number: SCXK 2012–0002).

### Lumbar Disc Herniation

LDH was conducted as previously described (24). A midline dorsal incision was made in pentobarbital-anesthetized (intraperitoneal injection, 50 mg/kg) rats, the midline dorsal muscles were separated along the L4–S1 spinous processes and the left L5 nerve roots and dorsal root ganglion (DRG) were exposed through laminectomy. Nucleus pulposus incised from the Co2–3 intervertebral disc was implanted next to the left L5 nerve root proximal to the DRG. Surgery was performed in sham rats without the nucleus pulposus implantation.

### Pain Behavior

The thermal withdrawal threshold was assessed as previously described (25); the assessment used a painful heat stimulus with a Model BME-410A Paw Stimulator Analgesia Meter (BME, Tianjin) that had a default latency period set at 5 min, and the maximum latency at the light intensity used was set at 20 s to avoid tissue damage. Each rat received five trials, with 10 min between each trial, in which each hindpaw was tested 30 s apart. The average latency until paw withdrawal was calculated.

The mechanical withdrawal threshold was tested as previously described (26), and it was assessed using a mechanical stimulation of the hindpaw with von Frey filaments (BME-403, Tianjin). Rats were placed in a clear box with a metal mesh floor for 15 min to habituate the rats to mechanical stimulation, and the plantar surface of the hindpaw was stimulated by adjusting the stiffness of the measurement probe beginning with 1 g of pressure (the stimulus intensities were 1, 2, 4, 6, 8, 10, 15, 26 g). Each stimulation persisted for 5–6 s, and 5 min intervals were used between stimulations to allow the animal to cease possible responding. Whenever, three positive responses to a given filament occurred with five stimulations, 50% probability thresholds of mechanical paw withdrawal were calculated. A positive sign of a withdrawal response was characterized as a rapid pulling back, biting, licking, or shaking of the stimulated hind limb.

Thermal withdrawal thresholds and mechanical withdrawal thresholds were assessed at 0, 2, 4, and 6 weeks after surgery by an investigator blinded to the treatment conditions.

### Depressive Behavior

A taste preference test (TPT) and a forced swim test (FST) were used to evaluate depressive behavior. The TPT was used to examine the behavior of anhedonia, and the FST was used to evaluate the state of despair, as previously described (27, 28). To examine chronic LBP-induced depressive behavior, we performed the FST and TPT at 0, 2, and 6 weeks after surgery.

The TPT was performed as previously described (27, 28). The animals were given free access to a standard rodent diet. Two bottles of 250 ml water were offered in their home cage on the first day of the experiment (habituation), but one of the bottles was replaced with 0.1% saccharin (#109185, Sigma) diluted in tap water on the next day (test). The test was initiated at 6.00 pm and ran for 24 h. TPT was expressed as a percentage of the volume of saccharin solution intake relative to the total liquid intake (saccharin solution plus regular water) over 24 h. Lower levels of preference for saccharin were indicative of anhedonia.

Subsequently, a modified version (27, 28) of the classic FST was conducted; the time was reduced to a single 5-min test, and higher immobility times indicated a state of despair. The rat was placed for 5 min in a glass container (60 cm in height and 30 cm in width) filled with tap water to a height of 45 cm and maintained at 22 to 25°C. The swimming behavior was videotaped and analyzed offline by an investigator blinded to the treatment conditions, and the total immobility time was calculated. Immobility was defined as not engaging in escape or exploratory behavior and consisted in moving the limbs just enough to keep the head above water. Rats were habituated in water for 5 min on the day before the formal test.

### FLX and Distilled Water Treatment

Fluoxetine hydrochloride (Sigma-Aldrich) was dissolved in distilled water and put on a shaker which kept the temperature at 37°C and was shaken gently for 30 min. It was administered at 20 mg/kg through gavage twice daily at 12-h intervals for 2 weeks starting from 4 weeks after surgery. Previous studies have demonstrated FLX effectively attenuated pain and depressive behavior at 18 mg/kg (29). Animals in distilled water treatment groups received the same volume of distilled water.

### Real-Time Reverse Transcriptase PCR

Total RNA was extracted from the hippocampus using TRIzol reagent (TaKaRa Bio, Dalian, China) according to the manufacturer's instructions. RNA was reverse transcribed to cDNA by using a reverse transcriptase kit (TaKaRa Bio, Dalian, China) on a Stratagene Robocycler Gradient 96 Thermal Cycler (Stratagene, California, USA). Real-time PCR assays were performed using a SYBR green Master Mix kit (Thermo Scientific, California, USA; Cat. No. #K0251) on an Mx3005 system (Stratagene). The following primers were used in this study: β-actin, (forward) 5′-GCA GGA GTA CGA TGA GTC CG-3′ and (reverse) 5′-ACG CAG CTC AGT AAC AGT CC-3′; TNF-α, (forward) 5′-GAC ACC ATG AGC ACG GAA AGC A-3′, and (reverse) 5′-CGC CAC GAG CAG GAA TGA GAA G-3′. The mRNA expression levels of the target gene were normalized to those of β-actin. Real-time PCR was done 6 weeks after surgery.

### Liquid Chromatography-Mass Spectrometry

#### Chemicals

Serotonin hydrochloride (#H9523) and high-performance liquid chromatography-grade methanol were purchased from Sigma.

#### Apparatus

Liquid chromatography-mass spectrometry (LC-MS) comprised a Prominence 20 A series UFLC System (Shimadzu, Kyoto, Japan) with a 100 × 2.1-mm Restek C18 Aqueous column (Restek, Pennsylvania, USA) and an API 4,000 Qtrap MS System (ABSciex, Massachusetts, USA). The LC-MS detection parameters and mobile phases were prepared as previously described (30).

#### Sample Preparation

The brain samples were prepared as previously described (28). Briefly, the hippocampus was dissected and crushed on ice and then homogenized in a 10-fold volume of a 0.1 M formic acid solution. The homogenates were centrifuged at 18,000 × g for 20 min at 4°C. The supernatants were collected and stored at −80°C until chromatographic analysis. The LC-MS detection was done 6 weeks after surgery.

#### Statistical Analysis

All the results are expressed as the means ± SEM. Statistical analysis was performed using Statistical Package for the Social Sciences (SPSS 19.0, Chicago, USA) software (independent samples *t*-test was used to compare the means of two independent samples, one-way analysis of variance was used for multiple comparisons). *P* < 0.05 was accepted as an index of statistically significant differences.

## Results

### Rats With LDH Shows Persistent Pain Behavior

**Figure 1:**
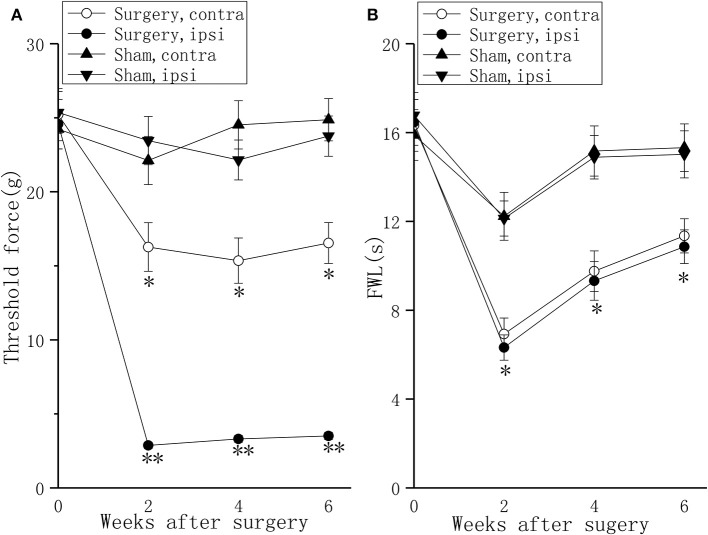
Behavioral responses in the mechanical withdrawal threshold test and thermal withdrawal latency test in a lumbar disc herniation model. The mechanical withdrawal threshold test **(A)** and the thermal withdrawal latency **(B)** on the ipsilateral foot and the contralateral foot were used to evaluate pain behavior at 2, 4, and 6 weeks after surgery. The data are presented as the means ± SEM, *n* = 10; *P < 0.05; **P < 0.01. FWL, foot withdrawal latency; ipsi, ipsilateral; contra, contralateral.

Compared to sham rats, the experimental group showed a significant drop on the ipsilateral foot and a moderate drop on the contralateral foot in mechanical withdrawal threshold at 2 and 4 weeks after surgery, and persisted for 6 weeks after surgery (Figure 1A, *P* < 0.01 or *P* < 0.05). Compared to sham rats, the experimental group showed drops on the bilateral feet in thermal withdrawal latency at 2 and 4 weeks after surgery, and persisted for 6 weeks after surgery (Figure 1B, *P* < 0.05).

### Consistent With the Persistent Pain, Rats With LDH Showed Chronic Depressive Behavior

**Figure 2:**
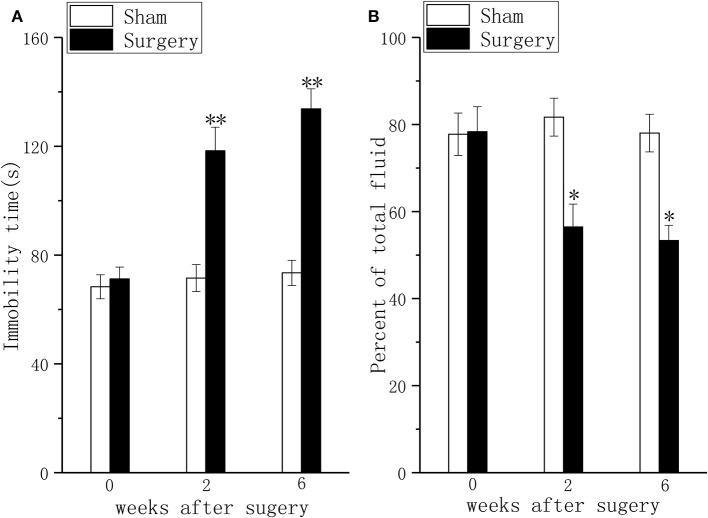
Behavioral responses in the forced swim test and the taste preference test in a lumbar disc herniation model. The duration of immobility during the forced swim test **(A)** and saccharin consumption **(B)** were used to evaluate depressive behavior at 2 and 6 weeks after surgery. The data are presented as the means ± SEM, *n* = 10; *P < 0.05; **P < 0.01.

Compared with sham rats, persistent pain induced depressive-like behavior in the FST (Figure 2A, *P* < 0.01) and the TPT (Figure 2B, *P* < 0.01) in rats with LDH at 2 weeks after surgery. At 6 weeks after surgery, the difference in FST (Figure 2A, *P* < 0.01) and TPT (Figure 2B, *P* < 0.01) between the sham group and the surgery group was significant.

### Consistent With the Behavior Alteration, TNF-α Expression Was Increased and 5-HT Concentration Was Decreased in Rats With Coexisting Chronic Pain and Depressive Behavior

**Figure 3:**
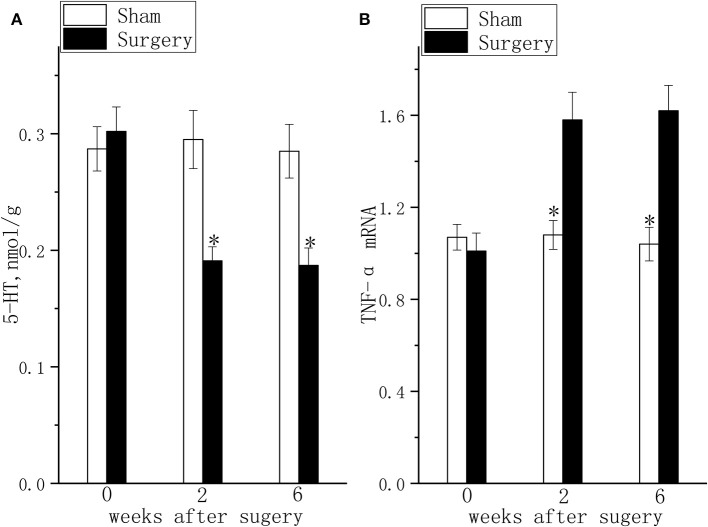
5-HT levels and TNF-α mRNA expression in the lumbar disc herniation model. The 5-HT concentration **(A)** and TNF-α mRNA expression **(B)** were detected at 2 and 6 weeks after surgery. The data are presented as the means ± SEM, *n* = 10; *P < 0.05.

The hippocampal TNF-α (Figure 3A, *P* < 0.05) mRNA levels were increased and hippocampal 5-HT concentration (Figure 3B, *P* < 0.05) was decreased at 2 weeks after surgery in rats with coexisting chronic pain and depressive behavior compared to those in the rats in the sham group. The difference in TNF-α mRNA (Figure 3A, *P* < 0.05) expression and 5-HT concentration (Figure 3B, *P* < 0.05) in the hippocampus of rats with coexisting chronic pain and depressive behavior was maintained for 6 weeks after surgery.

### Effect of FLX on Pain Behavior and Depressive Behavior

**Figure 4:**
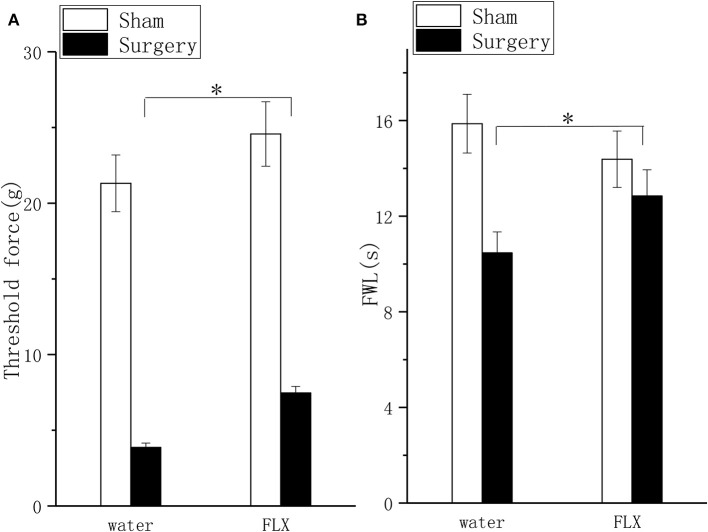
Effects of FLX on mechanical hyperalgesia and thermal hyperalgesia in a lumbar disc herniation model. The ipsilateral mechanical withdrawal threshold **(A)** and the ipsilateral thermal withdrawal latency **(B)** were used to evaluate pain behavior after 2 weeks of FLX and distilled water treatment (at 6 weeks after surgery). The data are presented as the means ± SEM, *n* = 10; *P < 0.05. FWL, foot withdrawal latency.

**Figure 5:**
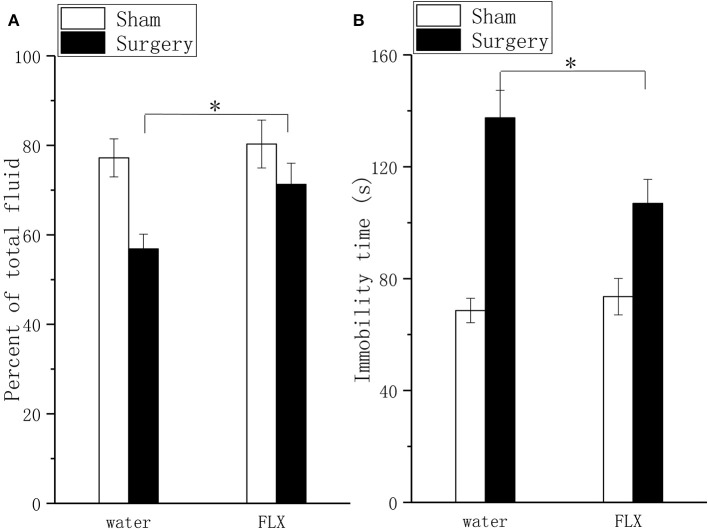
Effects of FLX on depressive behavior in the taste preference test and forced swim test in a lumbar disc herniation model. Saccharin consumption **(A)** and the duration of immobility during the forced swim test **(B)** were used to evaluate depressive behavior after 2 weeks of FLX and distilled water treatment (at 6 weeks after surgery). The data are presented as the means ± SEM, *n* = 10; *P < 0.05.

FLX, compared with water, produced significant but mild analgesic effects in the mechanical allodynia test (Figure 4A, *P* < 0.05) and thermal hyperalgesia test (Figure 4B, *P* < 0.05) after 2 weeks of treatment; FLX, compared with water, improved depressive behavior in the FST (Figure 5A, *P* < 0.05), and the TPT (Figure 5B, *P* < 0.05) after 2 weeks of treatment.

### Effect of FLX on 5-HT Concentration and TNF-α mRNA Expression

**Figure 6:**
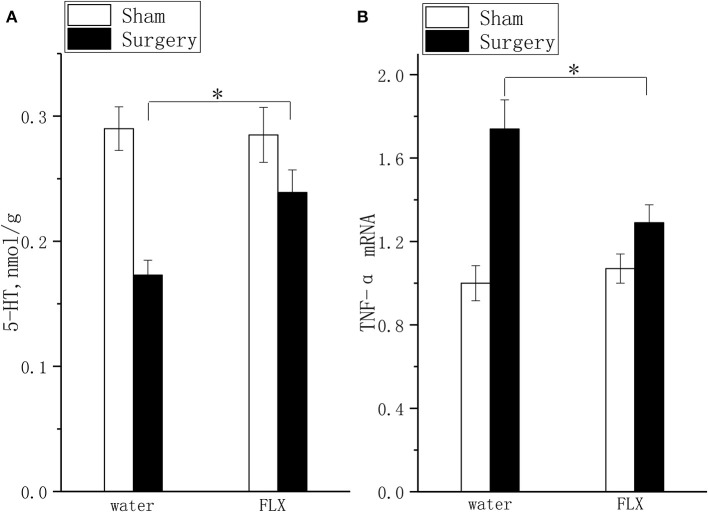
Effects of FLX on 5-HT concentration and TNF-α mRNA expression. The 5-HT concentration **(A)** and TNF-α mRNA expression **(B)** were detected after 2 weeks of FLX and distilled water treatment (at 6 weeks after surgery). The data are presented as the means ± SEM, *n* = 10; *P < 0.05.

FLX, compared with water, inhibited TNF-α mRNA expression (Figure 6A, *P* < 0.05) and elevated 5-HT (Figure 6B, *P* < 0.05) levels after 2 weeks of treatment.

## Discussion

Here, we demonstrated that rats with LDH showed persistent pain, which induced persistent depressive behavior. Consistent with the depressive behavior induced by persistent pain, TNF-α mRNA expression was elevated, and 5-HT was decreased in the hippocampus of rats with chronic pain. FLX effectively alleviated pain and depressive behavior through the inhibition of TNF-α mRNA expression and elevated 5-HT levels. These results indicated that rats with LDH showed behavioral and biochemical alterations that were in accordance with the characteristics of the comorbidity of pain and depression and were sensitive to FLX.

LDH is one of the most frequent causes of chronic LBP, but the mechanisms of depression in LBP patients are not completely understood. The establishment of an appropriate animal model is key to exploring the mechanisms. To our knowledge, no LBP model with depressive behavior has been established thus far. The present study provides the first evidence that chronic pain caused by LDH leads to depression, and this is the first study to verify the comorbidity of pain and depression in an LDH model in which behavioral alterations were similar to those observed in LDH patients.

In our study, nucleus pulposus application in the rat contributed to chronic thermal hyperalgesia and mechanical allodynia of both feet. These results are consistent with a previous study in which mechanical allodynia and thermal hyperalgesia also developed in the contralateral hindpaw (24). In fact, contralateral hyperalgesia has also been reported in other neuropathic pain models (31) and patients (32–34) and has been hypothesized to be mediated by a hypotenusal effect (34), migrated epidural fat (32), or prominent stenotic changes of the contralateral side (35).

We also found that LDH rats showed depressive behavior in the FST and TPT, and this depressive behavior was accompanied by pain and persisted for 6 weeks after surgery. The mechanism by which neuropathic pain arising in the spinal cord transmits nociceptive input to the thalamus through ascending pathways is complex. Many relay stations related to multidimensional emotional experiences play roles in this process. The hippocampus is one such important area that is involved in the formation and storage of memories associated with emotional events, affect, arousal and attention to pain and learning (36). Importantly, the neurochemical and morphological changes in the hippocampal network contribute to the comorbidity of pain and depression (37). Therefore, we assessed neurochemical changes in our study.

Consistent with the change in nociceptive and depressive behavior, TNF-α mRNA expression was elevated and 5-HT was lowered in the hippocampus of the LDH rats. These findings were consistent with previous reports that the downregulation of 5-HT and chronic neuroinflammation play key roles in the comorbidity of pain and depression (37). In fact, the behavioral and biochemical alterations are in accordance with the stress mechanism of the comorbidity of pain and depression (37). Persistent pain is a chronic stressor that induces central procytokine upregulation, and chronic neuroinflammation is further induced by oxidative stress (38), IDO activation and the kynurenine pathway (39). These factors lead to a deficit in 5-HT, which is considered one of the main reasons for depression (40, 41). Similar to its role in depression, central chronic neuroinflammation further activates afferent autonomic nerve pathways, decreases hippocampal 5-HT levels, decreases BDNF and contributes to persistent chronic pain (37, 42). In the present study, we did not explore the relationship between 5-HT levels and TNF-α expression because we only attempted to identify the biochemical alterations of the comorbidity of pain and depression.

SSRIs have been used to treat the comorbidity of pain and depression (13). They improve depressive behavior through inhibiting 5-HT reuptake, alleviating neuroinflammation—which further upregulates the 5-HT level—and decreasing the kynurenine derivatives that cause mitochondrial dysfunctions and neurotoxic effects in the CNS (43). The present study is supportive of those findings and showed that FLX increased 5-HT levels and inhibited TNF-α mRNA expression. These findings are consistent with previous reports that FLX improved depressive behavior partly through its anti-inflammatory effects (44, 45). As with the similar effect in depression treatment, FLX alleviated pain through its serotonergic antinociceptive effects and its intrinsic anti-inflammatory effect (22). The present results that the downregulated 5-HT level and increased TNF-α expression were normalized by FLX support the previous conclusion (22). Synthesizing the behavioral and biochemical alterations produced by FLX, the present study indicated that FLX attenuated pain and improved depressive behavior through the inhibition of 5-HT reuptake and anti-inflammatory effects. A previous study verified that FLX was inactive in mice with chronic constriction injury (46), which is why the models are different. Another study also found that FLX failed to attenuate mechanical hypersensitivity and depression-like behavior due to confounding pronociceptive actions at 5-HT_3_ receptors (47). However, in the present study, we found that FLX alleviated pain and depressive behavior, and the effect was moderate (*P* < 0.05 in the mechanical withdrawal threshold test, not *P* < 0.01). This result is consistent with previous reports that the analgesic effects of FLX are weak (29).

In fact, the analgesic effects and antidepressant effects of FLX were mild, which was consistent with the effects of FLX on TNF-α expression and 5-HT level. FLX has an anti-inflammatory effect, but the effect was moderate (22); the inflammation still existed, TNF-α expression was still high and the persistent low-grade inflammation decreased hippocampal 5-HT levels through many ways in accordance with previous descriptions, so the 5-HT level was still low. Considering the key roles of inflammation and 5-HT in the comorbidity of pain and depression, the mechanism by which persistent low-grade inflammation and low 5-HT level lead to residual pain and depressive behavior after FLX treatment could explain the results. After all, previous reports had verified that anti-inflammatory drugs markedly augmented the effects of FLX to alleviate pain and depressive behaviors (29).

Although the mechanism of the comorbidity of pain and depression is complex, we established an LDH model that showed behavioral and biochemical alterations that are in accordance with the characteristics of this comorbidity. This model may partly reflect the mechanism by which LDH patients develop depressive behavior, and the model verified the role of FLX in this comorbidity. The biochemical alterations should be further examined in LDH patients with depressive behavior. Although we verified the effect of FLX on pain, the mechanism by which FLX is moderately effective in attenuating pain and whether other medications could enhance these effects should be further investigated.

## Conclusion

The studies provide initial evidence that the LDH chronic pain model might serve as a model of the comorbidity of low back pain and depression. The finding that FLX improved depressive behavior and pain through normalized 5-HT concentrations and TNF-α mRNA expression establishes the initial mechanism of the comorbidity of pain and depression.

## Data Availability Statement

The datasets generated for this study are available on request to the corresponding author.

## Ethics Statement

The animals were housed and handled in strict accordance with the guidelines of the institutional and national Committees of Animal Use and Protection. The protocol was approved through the Committee on the Ethics of Animal Experiments of the Guangxi University of Chinese Medicine.

## Author Contributions

LC contributed to the study design, participated in the acquisition and analysis of data, the statistical analysis, and the manuscript drafting and revision. QH contributed to the study design. YL participated in the data analysis and manuscript revision. YuyH, WC, and LW contributed to the experimental implementation. The whole experiment was completed under the guidance of YueH.

### Conflict of Interest

The authors declare that the research was conducted in the absence of any commercial or financial relationships that could be construed as a potential conflict of interest.
